# Ryanodine receptors are targeted by anti-apoptotic Bcl-X_L_ involving its BH4 domain and Lys87 from its BH3 domain

**DOI:** 10.1038/srep09641

**Published:** 2015-04-15

**Authors:** Tim Vervliet, Irma Lemmens, Elien Vandermarliere, Elke Decrock, Hristina Ivanova, Giovanni Monaco, Vincenzo Sorrentino, Nael Nadif Kasri, Ludwig Missiaen, Lennart Martens, Humbert De Smedt, Luc Leybaert, Jan B. Parys, Jan Tavernier, Geert Bultynck

**Affiliations:** 1 KU Leuven, Laboratory of Molecular and Cellular Signaling, Department of Cellular and Molecular Medicine, B-3000 Leuven, Belgium; 2 University of Gent, Cytokine Receptor Lab, VIB Department of Medical Protein Research, B-9000 Gent, Belgium; 3 University of Gent, Computational Omics and Systems Biology Group, VIB Department of Medical Protein Research, B-9000 Gent, Belgium; 4 University of Gent, Physiology Group, Department of Basic Medical Sciences, B-9000 Gent, Belgium; 5 University of Siena, Molecular Medicine Section, Department of Molecular and Developmental Medicine, and Interuniversitary Institute of Myology, 53100 Siena, Italy; 6 Radboud University Medical Center, Donders Institute for Brain, Cognition and Behaviour, Department of Cognitive Neuroscience, Department of Human Genetics, 6500HB Nijmegen, The Netherlands

## Abstract

Anti-apoptotic B-cell lymphoma 2 (Bcl-2) family members target several intracellular Ca^2+^-transport systems. Bcl-2, via its N-terminal Bcl-2 homology (BH) 4 domain, inhibits both inositol 1,4,5-trisphosphate receptors (IP_3_Rs) and ryanodine receptors (RyRs), while Bcl-X_L_, likely independently of its BH4 domain, sensitizes IP_3_Rs. It remains elusive whether Bcl-X_L_ can also target and modulate RyRs. Here, Bcl-X_L_ co-immunoprecipitated with RyR3 expressed in HEK293 cells. Mammalian protein-protein interaction trap (MAPPIT) and surface plasmon resonance (SPR) showed that Bcl-X_L_ bound to the central domain of RyR3 via its BH4 domain, although to a lesser extent compared to the BH4 domain of Bcl-2. Consistent with the ability of the BH4 domain of Bcl-X_L_ to bind to RyRs, loading the BH4-Bcl-X_L_ peptide into RyR3-overexpressing HEK293 cells or in rat hippocampal neurons suppressed RyR-mediated Ca^2+^ release. *In silico* superposition of the 3D-structures of Bcl-2 and Bcl-X_L_ indicated that Lys87 of the BH3 domain of Bcl-X_L_ could be important for interacting with RyRs. In contrast to Bcl-X_L_, the Bcl-X_L_^K87D^ mutant displayed lower binding affinity for RyR3 and a reduced inhibition of RyR-mediated Ca^2+^ release. These data suggest that Bcl-X_L_ binds to RyR channels via its BH4 domain, but also its BH3 domain, more specific Lys87, contributes to the interaction.

The B-cell lymphoma 2 (Bcl-2) protein family has long been studied with respect to its prominent role in the regulation of apoptosis^1^,^2^. Beyond this, it is becoming increasingly clear that both the pro- and anti-apoptotic Bcl-2 family proteins are crucial regulators of intracellular Ca^2+^ signaling. In this way, Bcl-2 proteins affect various targets related to intracellular Ca^2+^ homeostasis^3^,^4^,^5^. More specific, this protein family was found to regulate the mitochondrial voltage-dependent anion channels^6^,^7^,^8^, plasma-membrane Ca^2+^-ATPases^9^, sarco/endoplasmic-reticulum Ca^2+^-ATPases (SERCA)^10^, Bax inhibitor 1^11^,^12^, inositol 1,4,5-trisphosphate (IP_3_) receptors (IP_3_R)^13^,^14^,^15^ and ryanodine receptors (RyRs)^16^.

Anti-apoptotic Bcl-2 proteins are characterized by the presence of four Bcl-2 homology (BH) domains important for their biological function^17^. Although their structural organization is very similar, Bcl-2 and Bcl-X_L_ may act in very different ways on their targets. As such, the BH4 domain of Bcl-2 is critical for binding to a site in the regulatory domain of the IP_3_R (a.a. 1389–1408 for mouse IP_3_R1) thereby inhibiting IP_3_-induced Ca^2+^ release^14^,^18^. In contrast, the BH4 domain of Bcl-X_L_ fails to bind to this IP_3_R domain and to inhibit IP_3_Rs^19^. Moreover, we showed that this difference between the BH4 domains of Bcl-2 and Bcl-X_L_ can largely be attributed to a single amino acid change (Lys17 in BH4-Bcl-2 corresponding to Asp11 in BH4-Bcl-X_L_) in the center of their respective BH4 domains. Indeed, the mutated BH4^K17D^ domain of Bcl-2 and mutated full-length Bcl-2^K17D^ are greatly impaired in targeting and regulating the IP_3_R.

We recently showed that, similar to its interaction with the IP_3_R, Bcl-2 via its BH4 domain targets a RyR region (a.a. 2263–2688 for mink RyR3) containing a highly conserved regulatory site (a.a. 2309–2330 for mink RyR3), which shows striking resemblance to the known Bcl-2 binding site on the IP_3_R^16^. The interaction of Bcl-2 and the RyR via its BH4 domain results in an inhibition of RyR-mediated Ca^2+^ release. The Bcl-2^K17D^ mutant does not show a dramatic loss of binding to the RyR and is as potent as wild-type Bcl-2 in inhibiting RyR-mediated Ca^2+^ release. These results may indicate that in contrast to the IP_3_R, which is differentially targeted by Bcl-2 and Bcl-X_L_, RyRs might have a common interaction site for both proteins and do not distinguish between these two proteins for their regulation.

In this paper, we show that similarly to Bcl-2, Bcl-X_L_ binds to the RyR via a site located in its central, modulatory domain, thereby inhibiting RyR-mediated Ca^2+^ release. Although the BH4 domain of Bcl-X_L_ was sufficient for inhibiting RyRs, we found that in full-length Bcl-X_L_ not only the BH4 domain but also the BH3 domain contributed to Bcl-X_L_/RyR-complex formation. In particular, we identified Lys87, located in the BH3 domain of Bcl-X_L_, as an important contributor of Bcl-X_L_ binding to the RyR.

## Results

### Bcl-X_L_ binds to RyR3

**Figure 1:**
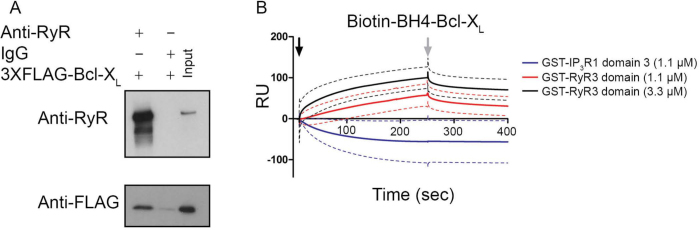
Bcl-X_L_ binds to a central regulatory region of RyR3. (A) Co-immunoprecipitation experiments were performed utilizing cell lysates from HEK RyR3 cells transiently overexpressing 3XFLAG-Bcl-X_L_. RyR3 was immunoprecipitated from these lysates utilizing a pan-RyR antibody. An anti-FLAG-HRP conjugated antibody was used for detecting co-immunoprecipitated 3XFLAG-Bcl-X_L_. Immunoblot showing the immunoprecipitated RyR3 (top) and co-immunoprecipitated 3XFLAG-tagged Bcl-X_L_ (bottom). Immunoprecipitations using non-specific IgG antibodies were applied as negative controls. All experiments were performed at least three times utilizing each time independently transfected cells and freshly prepared HEK RyR3 lysates. All samples were run using the same experimental conditions on the same gel/blot. The uncropped image is shown in Supplementary Fig. 1A. (B) Sensorgrams of the surface plasmon resonance experiments expressed in RU as a function of time. The biotin-BH4-Bcl-X_L_ peptide and the scrambled peptide were immobilized on different channels of a streptavidin-coated sensor chip. The channels on the chip were exposed to the indicated concentrations of purified GST-fusion proteins (GST-IP_3_R1 domain 3 and GST-RyR3 domain). Binding of the GST-tagged proteins to the scrambled peptides was subtracted from each sensorgram. GST-IP_3_R1 domain 3 bound stronger to the scrambled peptide than to the biotin-BH4-Bcl-X_L_ resulting in apparent negative values after this correction. The black arrow indicates the start of the association phase (addition of the GST-tagged proteins) and the grey arrow indicates the start of the dissociation phase (running buffer alone). Each sensorgram depicts the average of three experiments (full line) ± S.D. (dashed lines).

Bcl-2^K17D^ is a Bcl-2 mutant based on a critical difference between the BH4 domains of Bcl-2 and Bcl-X_L_ and is impaired in binding to and regulating IP_3_Rs^19^. However, this mutant still binds to and regulates RyRs with similar efficiencies as wild-type Bcl-2^16^, suggesting that Bcl-X_L_ may also bind to and regulate RyRs. Hence, we performed co-immunoprecipitation studies using lysates from HEK293 cells stably overexpressing RyR3 (HEK RyR3). In these cells, transiently overexpressed 3XFLAG-tagged Bcl-X_L_ co-immunoprecipitated with RyR3 indicating the formation of RyR3/Bcl-X_L_ complexes (Fig. 1A and Supplementary Fig. 1A for uncropped Western-blot images).

In our previous work we reported that the interaction between Bcl-2 and the RyR occurred via the BH4 domain of Bcl-2 and a central regulatory domain of the RyR (a.a. 22632263–2688 for mink 2688 for mink RyR3)^16^. To examine whether a direct interaction between RyRs and the BH4 domain of Bcl-X_L_ exists and whether this interaction occurs via the same or similar domains, surface plasmon resonance (SPR) experiments were performed (Fig. 1B). A concentration-dependent binding between biotin-BH4-Bcl-X_L_ immobilized to streptavidin coated SPR chips, and the purified GST-RyR3 domain (mink RyR3, a.a. 2263-2688) could be detected. In contrast, but consistent with our previous observations, purified GST-tagged IP_3_R1 domain 3 (mouse IP_3_R1, a.a. 9232263–2688 for mink 1581), which is known to bind to the BH4 domain of Bcl-2, failed to bind to biotin-BH4-Bcl-X_L_^19^. While biotin-BH4-Bcl-X_L_ was able to bind to the GST-RyR3 domain, it seemed to be less effective than biotin-BH4-Bcl-2^16^. To confirm the proper loading of the biotin-BH4-Bcl-X_L_ peptide to the sensor chip, we monitored the binding of an antibody directed against the BH4 domain of Bcl-X_L_, which caused a prominent increase in resonance unit (RU) values (Supplementary Fig. 2). Collectively, these results indicate that the interaction of Bcl-X_L_ with the RyR3 is direct and that Bcl-X_L_ via its BH4 domain targets the same domain as Bcl-2 on the RyR. However, the BH4 domain of Bcl-X_L_ seems to have a lower affinity for the GST-RyR3 domain compared to the BH4 domain of Bcl-2. This could indicate that biotinylation of the BH4 domain of Bcl-X_L_ influences its binding capabilities more than is the case for the BH4 domain of Bcl-2. Alternatively, other domains besides Bcl-X_L_'s BH4 domain may be involved in the interaction of full-length Bcl-X_L_ with the RyR. Therefore, we wanted to identify if other domains besides the BH4 domain of Bcl-X_L_ are important for interacting with the RyR.

### Superposition of the 3D-structures of Bcl-2 and Bcl-X_L_ reveals a spatial resemblance of Lys17 in the BH4 domain of Bcl-2 with Lys87 in the BH3 domain of Bcl-X_L_

**Figure 2:**
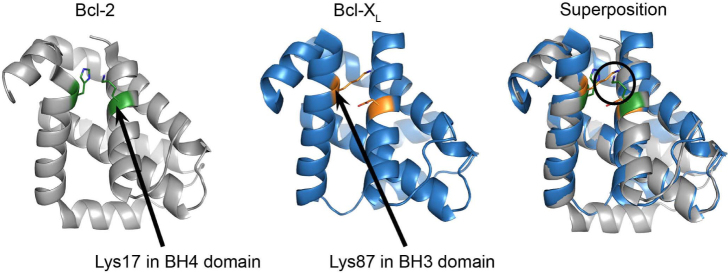
Spatial resemblance of Lys17 in the BH4 domain of Bcl-2 and Lys87 in the BH3 domain of Bcl-X_L_. Image showing the 3D-structures for Bcl-2 (left), Bcl-X_L_ (middle) and their *in silico* superposition (right). Lys17 in the BH4 domain of Bcl-2 and Lys87 in the BH3 domain of Bcl-X_L_ are indicated with arrows. The a.a. in green represent Lys17 and His94 in the BH4 and BH3 domain of Bcl-2 respectively. The a.a. in orange represent Asp14 and Lys87 in the BH4 and BH3 domains of Bcl-X_L_ respectively. The images were obtained by using PyMOL.

To identify the contribution and involvement of other Bcl-X_L_ domains for targeting RyR channels, an *in silico* superposition of the Bcl-2 (PDB-entry 4AQ3^20^) and Bcl-X_L_ (PDB-entry 1R2D^21^) structures was performed with the aid of PyMOL (The PyMOL Molecular Graphics System, Version 1.5.0.4 Schrödinger, LLC.). This superposition allowed the comparison of corresponding residues in the 3D-structures of Bcl-2 and Bcl-X_L_ (Fig. 2). This analysis revealed that the positively charged ε-amino terminus of the side chain of Lys87 in Bcl-X_L_, located in the BH3 domain, is in the same spatial constraints as the positively charged ε-amino terminus of the side chain of Lys17 located in the BH4 domain of Bcl-2. Furthermore, Lys87 did not seem to be part of the hydrophobic cleft of Bcl-X_L_, as it was directed towards the space facing the BH4 domain.

### The Bcl-X_L_^K87D^ mutant is impaired in RyR3 binding

**Figure 3:**
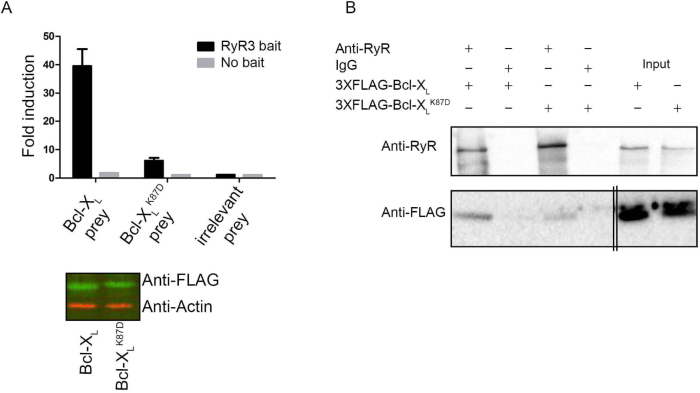
The Bcl-X_L_^K87D^ mutant is impaired in RyR3 binding. (A) Top: Representative example of a MAPPIT experiment. The binding is shown as fold induction value, calculated by dividing the average luciferase value of erythropoietin-stimulated cells by the average of non-stimulated cells. Binding of Bcl-X_L_ (Bcl-X_L_ prey), the Bcl-X_L_^K87D^ mutant (Bcl-X_L_^K87D^ prey) or irrelevant prey control (SV40 large T antigen) to the RyR3 domain (RyR3 bait) and as negative control the bait vector without RyR3 (No bait) are shown. Fold induction values at least 4 times higher than the irrelevant prey control are considered as *bona fide* protein-protein interactions. Values represent the average of three repeats within the same experiment ± S.D. All experiments were independently performed at least three times. Bottom: Odyssey Western blot analyses staining for the FLAG tag of the prey vector containing Bcl-X_L_ or the Bcl-X_L_^K87D^ mutant fusion proteins (green) or for actin (red) as a loading control. All samples were run using the same experimental conditions on the same gel/blot. The uncropped image is shown in Supplementary Fig. 1B. (B) Co-immunoprecipitations were performed in HEK RyR3 cells transiently overexpressing 3XFLAG-Bcl-X_L_ or 3XFLAG-Bcl-X_L_^K87D^ similarly as in Fig. 1A. Non-specific IgG antibodies were applied as negative controls. These experiments were performed at least three times utilizing each time independently transfected and freshly prepared HEK RyR3 cell lysates. All samples were run using the same experimental conditions and were derived from the same gel/blot, i.e. 3-8% tris-acetate gels for RyRs and 4-12% bis-tris gels for 3xFLAG-Bcl-X_L_. The double lines indicate that an additional empty lane separating the immunoprecipitated samples and the input samples was removed for the 3XFLAG-Bcl-X_L_ blot. The uncropped image is shown in Supplementary Fig. 1C.

The relevance of Lys87 in Bcl-X_L_ for RyR binding was addressed via mammalian protein-protein interaction trap (MAPPIT)^22^, an *in cellulo* protein-protein interaction assay. MAPPIT is based on the functional complementation of cytokine receptor signaling. To study the possible existence of RyR/Bcl-X_L_ complexes, the RyR3 domain was cloned downstream of a chimeric cytokine receptor (RyR3 bait), consisting of the extracellular domain of the erythropoietin (Epo) receptor fused to the transmembrane and cytosolic part of the leptin receptor. In the latter, three tyrosines were mutated to phenylalanine to down regulate receptor signaling. Bcl-X_L_ or the Bcl-X_L_^K87D^ mutant were cloned downstream of a part of the glycoprotein 130 receptor (Bcl-X_L_ or Bcl-X_L_^K87D^ prey). If the Bcl-X_L_ and Bcl-X_L_^K87D^ prey constructs interact with the RyR3 bait construct, functional complementation of the chimeric cytokine receptor occurs, leading to ligand-dependent downstream STAT signaling. The latter is monitored via a luciferase reporter assay driven by a STAT-sensitive promoter. We also used the SV40 large antigen T (irrelevant prey) as a prey to monitor the signal representing the non-specific binding to RyR3. As a negative control, binding of the chimeric cytokine receptor without the RyR3 fragment (no bait) to the two Bcl-X_L_ preys was also assessed. These MAPPIT results confirmed the data obtained via SPR and co-immunoprecipitation experiments, showing that Bcl-X_L_ could interact with the RyR3 domain in a cellular context (Fig. 3A, top). Moreover, the Bcl-X_L_^K87D^ mutant was severely impaired in interacting with the RyR3 domain without affecting its expression (Fig. 3A, bottom panel and Supplementary Fig. 1B for uncropped Western-blot images). No binding was detected when the RyR3 domain was not present in the bait vector (Fig. 3A, top panel), indicating that the interaction was specific.

The impact of mutating Lys87 into Asp was also examined in the context of the full-length RyR3 protein using co-immunoprecipitation experiments. Consistent with the MAPPIT data, 3XFLAG-tagged Bcl-X_L_^K87D^ displayed a reduced affinity for full-length RyR3 channels (Fig. 3B and Supplementary Fig. 1C for uncropped Western-blot images).

Taken together, these data indicate that Bcl-X_L_, similarly to Bcl-2, binds via its BH4 domain to the same regulatory domain on RyR3. However, whereas for Bcl-2 the BH4 domain appears to be the main determinant for complex formation with RyR channels, it seems that for Bcl-X_L_ both the BH4 domain and the BH3 domain, likely via Lys87, contribute to the interaction with RyR channels.

### Bcl-X_L_, but not Bcl-X_L_^K87D^, inhibits RyR3-mediated Ca^2+^ release

**Figure 4:**
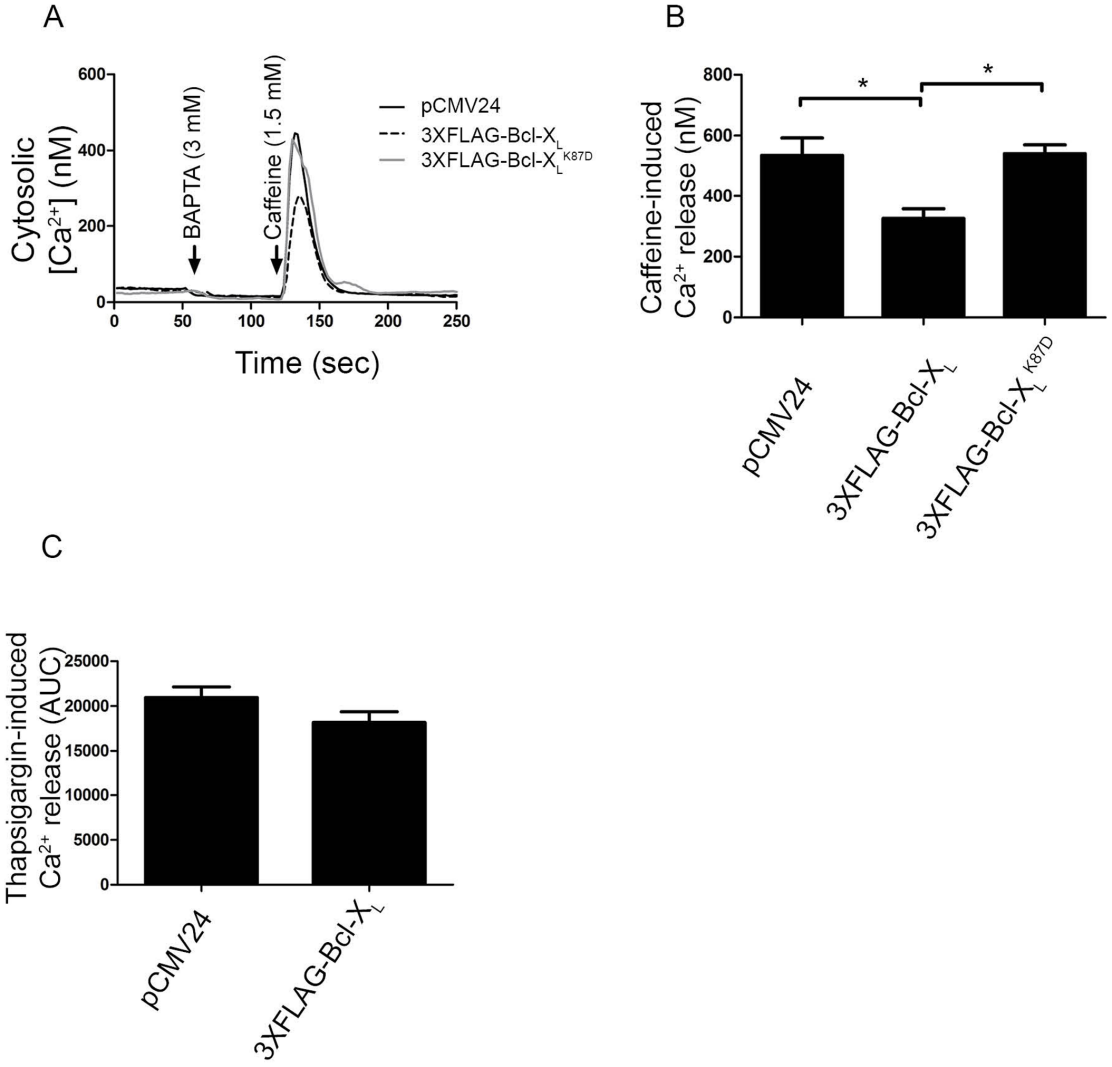
Bcl-X_L_ but not Bcl-X_L_^K87D^ inhibits RyR-mediated Ca^2+^ release. Single-cell cytosolic [Ca^2+^] measurements were performed in HEK RyR3 cells utilizing Fura-2-AM. (A) Average calibrated [Ca^2+^] trace of 15 to 20 HEK RyR3 cells transfected (mCherry positive) with an empty vector as control (pCMV24), 3XFLAG-Bcl-X_L_ or 3XFLAG-Bcl-X_L_^K87D^. Addition of BAPTA and caffeine is indicated by the arrows. (B) Quantitative analysis of the single-cell cytosolic [Ca^2+^] measurements. Values indicate averages of all peak values ± S.E.M. These experiments were independently performed at least four times (>120 cells/condition) (p = 0.008). (C) Quantitative analysis of the ER Ca^2+^-store content. ER-store content was determined by performing similar experiments as in A except that 1 μM thapsigargin was used as the stimulus. The values indicate the average area under the curve (AUC) ± S.E.M. of at least three independent experiments (>80 cells/condition).

Driven by the fact that Bcl-X_L_ can bind to RyR3, we examined whether Bcl-X_L_ could modulate RyR-mediated Ca^2+^ release (Fig. 4). Single-cell cytosolic [Ca^2+^] measurements in HEK RyR3 cells loaded with Fura-2-AM were performed (Fig. 4A). An empty vector (pCMV24) control, 3XFLAG-tagged Bcl-X_L_ or the 3XFLAG-tagged Bcl-X_L_^K87D^ mutant were transiently transfected into the HEK RyR3 cells. An mCherry coding plasmid was co-transfected (at a 1:3 ratio) to identify transfected cells. After chelating extracellular Ca^2+^ with BAPTA (3 mM), caffeine (1.5 mM) was applied to induce RyR-mediated Ca^2+^ release. Overexpression of 3XFLAG-tagged Bcl-X_L_ inhibited caffeine-induced Ca^2+^ release compared to the empty vector control. The Bcl-X_L_^K87D^ mutant failed to inhibit caffeine-induced Ca^2+^ release (Fig. 4B), correlating with its poor RyR3-binding properties. To exclude that the observed reduction in caffeine-induced Ca^2+^ release upon Bcl-X_L_ overexpression would have been due to an indirect effect via lowering of the Ca^2+^-filling state of the endoplasmic reticulum (ER), we determined the amount of thapsigargin (1 μM)-releasable Ca^2+^. This irreversible SERCA inhibitor causes a depletion of the ER Ca^2+^ stores and provides a good measure for the ER Ca^2+^-store content. The ER Ca^2+^-store content was not affected by overexpression of 3XFLAG-tagged Bcl-X_L_ (Fig. 4C). This supports the view that Bcl-X_L_, similarly to Bcl-2, suppresses RyR-mediated Ca^2+^ release.

### The BH4 domain of Bcl-X_L_ by itself seems sufficient to inhibit RyR-mediated Ca^2+^ release

**Figure 5:**
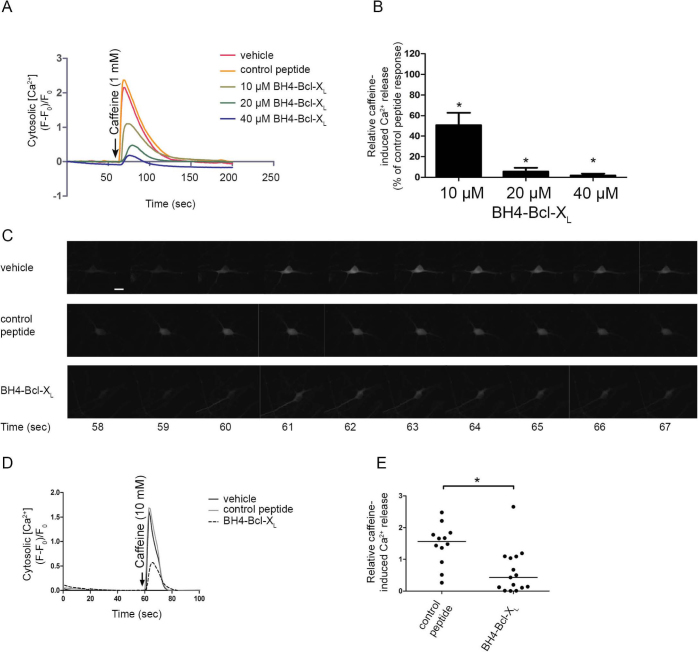
The BH4 domain of Bcl-X_L_ by itself was sufficient to inhibit RyR-mediated Ca^2+^ release. (A) Representative trace of the performed Fluo-3-AM single-cell cytosolic [Ca^2+^] measurements in HEK RyR3 cells loaded by electroporation with either the vehicle (DMSO), a control peptide or the BH4 domain of Bcl-X_L_. The addition of caffeine is indicated by the arrow. Traces were normalized to the baseline fluorescence ((F-F_0_)/F_0_). (B) Quantitative analysis of the single-cell cytosolic [Ca^2+^] measurements with indicated concentrations of the BH4 domain of Bcl-X_L_. Values indicate caffeine-induced Ca^2+^ release after electroporation loading with different concentrations of the BH4 domain of Bcl-X_L_ relative to the response after electroporation loading with the same concentration of the control peptide. Values depict average ± S.E.M. of at least four independent experiments (p-values were 0.0037, 0.001 and 0.0039 for 10 μM, 20 μM and 40 μM of the BH4 domain of Bcl-X_L_ respectively). (C-E) Single-cell [Ca^2+^] measurements performed in 14- to 18-day-old hippocampal cultures. GCaMP3, introduced into these neurons via adeno-associated infection, was used as cytosolic Ca^2+^ indicator. Utilizing whole-cell voltage clamp the membrane potential of the neurons was clamped at −60 mV. 20 μM of the BH4 domain of Bcl-X_L_, a control peptide or the vehicle (DMSO) was introduced into each measured neuron via the patch pipette. All experiments were performed in the presence of 1 μM tetrodotoxin. A 10 mM caffeine puff was locally administered via a second patch pipette positioned 15-25 μm from the soma of the neuron. (C) Time lapse of a typical experiment for each of the tested conditions. Caffeine was administered after 60 sec. The scale bar depicts 5 μm. (D) Typical responses to caffeine after loading the neurons with 20 μM of either the control peptide the BH4 domain of Bcl-X_L_ or the vehicle. Traces were normalized to the baseline fluorescence ((F-F_0_)/F_0_). The arrow indicates when caffeine was administered. (E) Scatter plot showing peak responses of all performed measurements and the median (horizontal line). All values were normalized to the caffeine response after vehicle control treatment (p = 0.0037, N = 12 and N = 15 for the control peptide and BH4-Bcl-X_L_ respectively).

In order to assess whether the BH4 domain of Bcl-X_L_ is sufficient for inhibiting RyR-mediated Ca^2+^ release, Fluo-3-AM loaded HEK RyR3 cells were loaded acutely with the BH4 domain of Bcl-X_L_, a control peptide or the vehicle via electroporation (Fig. 5A). The BH4 domain of Bcl-X_L_, but not a control peptide, suppressed caffeine (1 mM)-induced Ca^2+^ release. The BH4 domain of Bcl-X_L_ inhibited caffeine-induced Ca^2+^ release in a concentration-dependent manner (Fig. 5B). This indicates that the BH4 domain of Bcl-X_L_ was sufficient for inhibiting RyR-mediated Ca^2+^ release.

We also assessed whether the BH4 domain of Bcl-X_L_ could inhibit endogenous RyR channels by using 14- to 18-day-old rat hippocampal cultures known to express different RyR isoforms^23^. The experimental set-up was identical to the one previously used for characterization of the effect of the BH4 domain of Bcl-2 on native RyRs^16^. Cytosolic [Ca^2+^] was monitored in GCaMP3-expressing hippocampal neurons. The BH4 domain of Bcl-X_L_, a control peptide or the vehicle were introduced into the neurons via a patch pipette. After loading the neuron for five minutes with the peptides or vehicle, cytosolic [Ca^2+^] measurements were started. RyR-mediated Ca^2+^ release was triggered via a local puff of caffeine (10 mM) delivered via a second patch pipette positioned next to the neuron. A time lapse (Fig. 5C) and a [Ca^2+^] trace (Fig. 5D) of a typical experiment are shown for each condition. Loading of the neurons with the BH4 domain of Bcl-X_L_ (20 μM) caused a significant reduction of the caffeine-induced Ca^2+^ release compared to the control peptide (Fig. 5 D, E). These results indicate that the BH4 domain of Bcl-X_L_ can regulate endogenously expressed RyR channels.

### Bcl-X_L_ and its BH4 domain directly inhibit RyRs at the level of the ER

**Figure 6:**
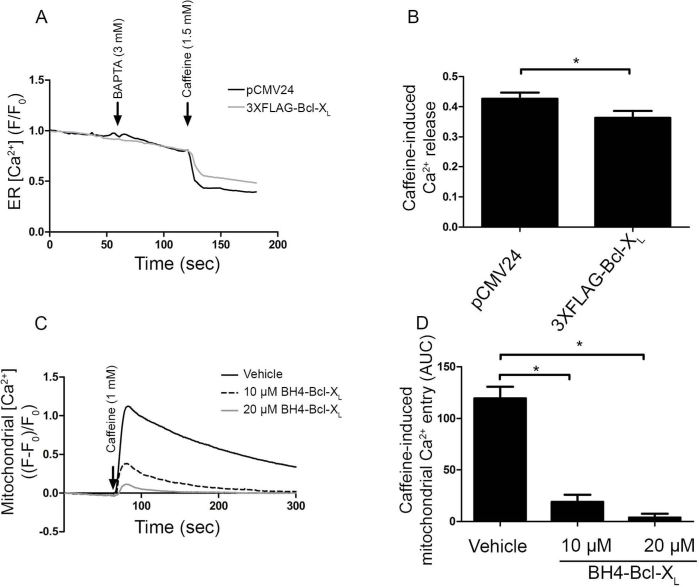
Bcl-X_L_ and its BH4 domain directly inhibit RyR-mediated Ca^2+^ release from the ER. (A) Typical average normalized (F/F_0_) traces of single-cell ER [Ca^2+^] measurement performed in HEK RyR3 cells transfected with G-CEPIA1er plasmid. G-CEPIA1er-positive cells transfected with the empty control vector (pCMV24) or 3XFLAG-Bcl-X_L_ were selected for these measurements. After chelating extracellular Ca^2+^ with BAPTA, caffeine was added to stimulate RyR-mediated Ca^2+^ release (arrows). (B) Quantitative analysis of the performed experiments. For each trace the caffeine-induced Ca^2+^ release was determined by subtracting the fluorescence after caffeine addition (during plateau phase) from the fluorescence just before caffeine addition after normalization. Values depict average ± S.E.M. These experiments were independently repeated at least four times (>100 cells/condition) (p = 0.0018). (C) Normalized ((F-F_0_)/F_0_) representative traces of mitochondrial [Ca^2+^] measurements. The vehicle (DMSO) or the BH4 domain of Bcl-X_L_ (10 μM and 20 μM) were introduced into Rhod-FF-loaded HEK RyR3 cells via electroporation loading. Mitochondrial Ca^2+^ was measured after caffeine (arrow) stimulation. (D) Quantification of the performed experiments. Values show the average caffeine-induced mitochondrial Ca^2+^ entry as area under the curve (AUC) ± S.E.M. Experiments were independently performed at least three times (p<0.0001).

Bcl-X_L_ and its isolated BH4 domain as a synthetic peptide inhibit the caffeine-induced [Ca^2+^] rise in the cytosol. Bcl-X_L_ has also been implicated in the control of mitochondrial Ca^2+^ transport at the level of VDAC1. Bcl-X_L_ was shown to inhibit Ca^2+^ uptake into the mitochondria^6^,^24^. However, it was also reported that Bcl-X_L_ could stimulate mitochondrial Ca^2+^ uptake^25^. The latter effect could result in a decrease in caffeine-induced [Ca^2+^] rise in the cytosol. Therefore, we set out to document whether the decrease in caffeine-induced Ca^2+^ release in the cytosol by Bcl-X_L_ is due to a decreased Ca^2+^ release from the ER or to an increased Ca^2+^ accumulation into the mitochondria. Direct ER-Ca^2+^ measurements were performed in HEK RyR3 cells utilizing a recently described green fluorescent CEPIA1 protein, that is targeted to the lumen of the ER (G-CEPIA1er)^26^. HEK RyR3 cells were transiently transfected with the empty vector (pCMV24) as control or with 3XFLAG-tagged Bcl-X_L_ in combination with the G-CEPIA1er-encoding vector (at a 3:1 ratio). G-CEPIA1er-positive cells were selected and measurements were performed as in Fig. 4A. A typical average trace of one experiment and the quantification of all performed experiments are shown in Fig. 6A and B, respectively. These results indicate that overexpression of 3XFLAG-Bcl-X_L_ suppressed the caffeine-induced Ca^2+^ release from the ER, supporting a model in which the inhibitory effect of Bcl-X_L_ on RyR-mediated [Ca^2+^] rise in the cytosol occurs at least in part due to inhibition of the Ca^2+^ release from the ER. Finally, we set out to directly measure the effect of the BH4 domain of Bcl-X_L_ on caffeine-induced mitochondrial Ca^2+^ entry. Rhod-FF-loaded HEK RyR3 cells were electroporated with either the vehicle (DMSO) or the BH4 domain of Bcl-X_L_ (10 and 20 μM) and then stimulated with caffeine. Caffeine stimulation resulted in an increase in mitochondrial [Ca^2+^] (Fig. 6C). Compared to the vehicle control however, the BH4 domain of Bcl-X_L_ potently inhibited the mitochondrial Ca^2+^ entry (Fig. 6 C, D). Furthermore, the effectiveness of BH4-Bcl-X_L_ to inhibit caffeine-induced [Ca^2+^] rise in the mitochondria seemed higher than for inhibiting the caffeine-induced [Ca^2+^] rise in the cytosol, because 10 μM BH4-Bcl-X_L_ inhibited caffeine-induced Ca^2+^ release in the cytosol by about 50% but inhibited caffeine-induced Ca^2+^ uptake in the mitochondria by about 90%. Taken together these data suggest that BH4-Bcl-X_L_ likely inhibits, rather than stimulates, mitochondrial Ca^2+^ accumulation. This is consistent with our recent findings showing that BH4-Bcl-X_L_ directly interacts with VDAC1 and suppressed VDAC1-mediated Ca^2+^ transfer into the mitochondria^27^. These experiments indicate that Bcl-X_L_ can directly inhibit the caffeine-induced Ca^2+^ release at the level of the ER and potently inhibit mitochondrial Ca^2+^ uptake under these experimental settings. We therefore conclude that the observed decrease in caffeine-induced Ca^2+^ release in the cytosol (Fig. 4 and 5) is mainly due to a direct inhibition of RyR3.

## Discussion

The main conclusion of this paper is that Bcl-X_L_ binds to and regulates RyR3 channels. Similarly to Bcl-2, Bcl-X_L_ targets the central modulatory domain of the RyR protein, thereby suppressing RyR-mediated Ca^2+^ release. Moreover, the BH4 domain of Bcl-X_L_ was sufficient to inhibit both over- and endogenously expressed RyR channels in HEK293 cells or primary rat hippocampal neurons respectively. Consistent with this, the BH4 domain of Bcl-X_L_ could bind to the purified RyR3 domain. However, the RyR3-binding efficiency of the BH4 domain of Bcl-X_L_ seemed much lower than that of the BH4 domain of Bcl-2. Via an *in silico* superposition of the Bcl-2 and Bcl-X_L_ crystal structures, a spatial overlap was observed between Lys17 in the BH4 domain of Bcl-2 and Lys87 in the BH3 domain of Bcl-X_L_: the positively charged ε-amino groups of their side chains coincide in space. Consistent with the moderate RyR3-binding properties of the isolated BH4 domain of Bcl-X_L_, we found that Lys87 from Bcl-X_L_ played a prominent role in binding to and regulating RyR3.

The association of Bcl-X_L_ with RyR channels and its functional implications appear to be very similar as the ones observed for Bcl-2, since i) RyR3/Bcl-X_L_ binding is direct; ii) the binding of Bcl-X_L_ to RyR3 occurs, at least in part, via the BH4 domain; iii) Bcl-X_L_ overexpression inhibits RyR-mediated Ca^2+^ release; and iv) the BH4 domain of Bcl-X_L_ is also sufficient to suppress RyR activity. These findings correlate with the fact that the Bcl-2^K17D^ mutant and BH4-Bcl-2^K17D^ remain capable of binding to and regulating RyR channels, although this mutation changes the lysine critical for binding to the IP_3_R into the Asp11 residue in the BH4 domain of Bcl-X_L_^16^. This lack of selectivity between Bcl-2 and Bcl-X_L_ may illustrate an important difference between IP_3_R- and RyR-mediated Ca^2+^ release. However, the binding of Bcl-X_L_ versus Bcl-2 to RyRs in native tissues expressing RyRs ought to be further explored. In particular, it will be important to carefully analyze the Bcl-2- and Bcl-X_L_-expression levels in the relevant tissues and to determine whether a preferential binding of Bcl-2 or Bcl-X_L_ to RyR channels exists in cells expressing both Bcl-2 and Bcl-X_L_. Despite these similarities, the molecular determinants underlying RyR/Bcl-X_L_-complex formation do not seem identical to those of Bcl-2, because the BH4 domain of Bcl-X_L_ by itself displays rather moderate RyR3-binding properties. As a consequence, additional domains seem to be involved in RyR/Bcl-X_L_-complex formation. Here, we identified Lys87, located in the BH3 domain of Bcl-X_L_, as a critical determinant contributing to binding to and regulating RyR channels. Despite the importance of Lys87, the BH4 domain of Bcl-X_L_ alone was able to suppress RyR activity.

The BH4 domain of Bcl-X_L_ has been implicated in numerous studies to display strong anti-apoptotic and protective effects against a wide variety of insults and triggers, including in the heart^28^,^29^,^30^, endothelial cells^31^,^32^, blood cells^33^,^34^,^35^, pancreatic islets^36^ and neurons^37^. Many of the cell types and tissues reported to benefit from the BH4 domain of Bcl-X_L_ for their survival endogenously express RyR channels (cardiomyocytes, lymphocytes, pancreatic islets and neurons). Furthermore, in many apoptotic paradigms, reactive oxygen species (ROS) are implicated. ROS can impact the redox state and activity of the RyR channels (reviewed by Ref. 38). Mild increases in ROS moderately increase RyR activity by increasing its sensitivity for Ca^2+^ 39. However, severe ROS production associated with oxidative stress (e.g. in the context of ischemia/reperfusion injury) can lead to a continuously opening of the RyR channels, provoking an excessive Ca^2+^ leak from the ER or sarcoplasmic reticulum^40^. In the context of the heart, ROS has been clearly implicated to cause unzipping of the interdomain interactions critical for RyR2-channel stabilization^41^,^42^. During oxidative stress conditions, the BH4 domain of Bcl-X_L_ may thus inhibit excessive RyR-mediated Ca^2+^ release from the intracellular Ca^2+^ stores in addition to exerting its protective effects at the mitochondria, thereby providing additional protection against cell death.

RyRs have important physiological functions in a variety of excitable cells and tissues, including skeletal muscle, cardiac muscle, neurons and pancreatic cells^43^,^44^,^45^,^46^. Furthermore, dysregulation of RyRs, either by somatic mutations or by altered expression levels, has been implicated in a variety of pathophysiological conditions, including malignant hyperthermia and central core disease^47^,^48^, cardiac diseases^49^,^50^,^51^ and neurodegenerative diseases like Alzheimer's disease^52^,^53^,^54^ and Huntington's disease^55^. At this point, the existence and physiological relevance of RyR/Bcl-2- and RyR/Bcl-X_L_-complex formation in these tissues and their potential disturbance in RyR-associated pathophysiologies will require further research.

In conclusion, our data further expand the number of Bcl-2-family members that are able to form protein complexes with RyR channels, thereby underpinning their critical role in regulating intracellular Ca^2+^ dynamics at the level of intracellular Ca^2+^-release channels.

## Methods

### Chemicals, antibodies and peptides

Unless otherwise specified, all chemicals were purchased from Sigma-Aldrich (St. Louis, MO, USA). The following antibodies were used: mouse monoclonal anti-actin antibody, anti-FLAG M2 antibody and HRP-conjugated anti-FLAG M2 antibody (Sigma-Aldrich), mouse monoclonal anti-RyR antibody 34C (Thermo Scientific, Rockford, IL, USA, or Developmental Studies Hybridoma Bank, University of Iowa, Iowa, USA) and mouse monoclonal anti-Bcl-X_L_ antibody YTH-2H12 (Trevigen, Gaithersburg, WV, USA). The sequences of the peptides used in this study were:

Biotin-BH4-Bcl-X_L_: Biotin-MSQSNRELVVDFLSYKLSQKGYSW (also used without the biotin tag)

Biotin-scrambled BH4-Bcl-X_L_: Biotin-WYSKQRSLSGLVMYVLEDKNSQFS

Control peptide: WYEKQRSLHGIMYYVIEDRNTKGYR

These peptides were synthesized by Life Tein (Hillsborough, NJ, USA) with a purity of at least 85%.

### Plasmids, constructs and protein purifications

3XFLAG-Bcl-X_L_ was obtained as previously described^19^. The 3XFLAG-Bcl-X_L_^K87D^ mutant was obtained by PCR site-directed mutagenesis utilizing the following primers: forward: 5′ATCCCCATGGCAGCAGTAGATCAAGCGCTGAGGGAGGCA3′, and reverse: 5′TGCCTCCCTCAGCGCTTGATCTACTGCTGCCATGGGGAT3′. The pCMV G-CEPIA1er containing plasmid was a gift from Dr. Masamitsu Iino (Addgene plasmid # 58215)^26^. The GST-IP_3_R1 domain 3 construct and the GST-RyR3 construct were obtained and purified as described^16^.

### Cell culture, transfections and dissociated hippocampal cultures

All media and supplements added to the medium used in this paper were purchased from Life Technologies (Ghent, Belgium). HEK293 cells stably overexpressing RyR3 were cultured at 37°C in a 5% CO_2_ incubator in α-Minimum Essential Medium supplemented with 10% fetal calf serum, 100 IU/mL penicillin, 100 μ*g*/mL streptomycin, 2 mM glutamax and 800 μg/mL G418^56^. HEK293 cells were grown in Dulbecco's Modified Eagle Medium containing 4500 mg/L glucose, 10% fetal bovine serum and 50 μg/mL gentamicin^57^.

24 hours after seeding, the 3XFLAG-Bcl-X_L_ or the 3XFLAG-Bcl-X_L_^K87D^ mutant construct were introduced into the HEK RyR3 cells utilizing JETPrime transfection reagent (Polyplus Transfections, Illkirch, France) according to the manufacturer's protocol. 48 hours later the cells were harvested and lysed utilizing a CHAPS-based lysis buffer (pH 7.5, 50 mM Tris-HCl, 100 mM NaCl, 2 mM EDTA, 50 mM NaF, 1 mM Na_3_VO_4_, 1% CHAPS and protease inhibitor tablets (Roche, Basel, Switzerland)). For single-cell cytosolic [Ca^2+^] measurements the same constructs or the empty pCMV24 vector were introduced 48 hours after seeding in the HEK RyR3 cells utilizing X-tremeGENE HP DNA transfection reagent (Roche) according to the manufacturer's protocol. A pcDNA 3.1(-) mCherry expressing vector was co-transfected at a 1:3 ratio as a selection marker. For direct ER [Ca^2+^] measurements, the G-CEPIA1er construct was co-transfected (ratio 3:1) and used as selection marker instead of the mCherry expressing vector. Dissociated hippocampal cultures were obtained as described previously^58^. All animal experiments were performed according to approved guidelines.

### SPR analysis

SPR analysis was performed using a Biacore T200 (GE Healthcare, Diegem, Belgium). Immobilization to the streptavidin-coated sensor chip (BR-1005-31; GE Healthcare) and SPR measurements were performed as described previously^19^. NaOH (50 mM) with 0.0026% SDS was used as a regeneration buffer.

### Immunoblot analysis

Samples were prepared and used as previously described^19^. For visualization of RyRs, NuPAGE 3–8% tris-acetate gels were run. Detection was performed using Pierce ECL Western Blotting Substrate (Thermo Scientific) when using the Chemidoc™ MP system (Bio-Rad, Nazareth Eke, Belgium) or an X-OMAT 1000 processor (Kodak, Zaventem, Belgium). When using the Odyssey imager (Westburg, Leusden, The Netherlands) detection was performed using anti-mouse-IRDye800 (green) or anti-rabbit-IRDye700 (red) as secondary antibodies (Thermo Scientific).

### Co-immunoprecipitation experiments

Co-immunoprecipitation experiments were performed utilizing a co-immunoprecipitation kit (Thermo Scientific). RyR antibody or mouse IgG control antibody (Santa Cruz Biotechnology, Heidelberg, Germany) was immobilized according to the manufacturer's protocol. Gelatine was removed from the IgG control antibody utilizing a Pierce Antibody Clean-up Kit (Thermo scientific). Precleared HEK RyR3 lysates containing the 3XFLAG-Bcl-X_L_ constructs (150 μg) were added to the resin to which the antibodies were immobilized and allowed to incubate overnight at 4°C. The next day, the resin was washed at least five times utilizing the CHAPS-based lysis buffer. The immune complexes were eluted by boiling (95°C) in 50 μL 2× LDS (Life Technologies) supplemented with 1/200 β-mercaptoethanol for 5 min.

### MAPPIT

The RyR3 domain was amplified by PCR using the following primers, forward: 5′TAGTTGTCGACGAAGAGAGAAGTCATGGAGGA3′, and reverse: 5′TAGTTGCGGCCGCCTATTTGGTCCTCTCCACA3′, and cloned in the pSEL+2L bait vector^59^, using the restriction enzymes SalI and NotI. Bcl-X_L_ was cloned in the pMG1-GW plasmid (prey vector)^22^ using the Gateway recombination technology as described by the manufacturer (Life Technologies). Utilizing the same primers as described before, the Bcl-X_L_^K87D^ mutation was also introduced in this construct via site directed mutagenesis. The MAPPIT analyses were done as previously described^22^ with minor changes. Briefly, HEK293 cells were seeded in 96-well plates. Six wells per condition were transfected with the different combinations of bait, prey and reporter plasmid (rPAP1-luci) using the calcium phosphate method. The next day, half of the wells were stimulated with 5 ng/mL Epo while the other half were left untreated. 24 hours later the cells were lysed and after the addition of substrate the luciferase activity was determined using a luminometer. The fold induction was obtained by dividing the average value of the stimulated cells by the average value of the non-stimulated cells.

### Electroporation loading

Electroporation loading of HEK RyR3 cells was performed as previously described^16^,^60^.

### Single-cell cytosolic Ca^2+^ imaging

Fura-2-AM and Fluo-3-AM [Ca^2+^] measurements in HEK RyR3 cells and GCaMP3 single-cell [Ca^2+^] measurements in dissociated hippocampal neurons were performed as previously described^16^.

### Single-cell ER Ca^2+^ imaging

The G-CEPIA1er construct was introduced into HEK RyR3 cells as described above. A Zeiss Axio Observer Z1 Inverted Microscope equipped with a 20× air objective and a high-speed digital camera (Axiocam Hsm, Zeiss, Jena, Germany) were used for these measurements. Changes in fluorescence were monitored in the GFP channel (480/520 excitation/emission). To chelate extracellular Ca^2+^, 3 mM BAPTA (Alfa Aesar, Ward Hill, MA, USA) was added. One minute later 1.5 mM caffeine was added to trigger RyR-mediated Ca^2+^ release. All traces were normalized (F/F_0_) where F_0_ is the starting fluorescence of each trace.

### Single-cell mitochondrial Ca^2+^ imaging

HEK RyR3 cells were loaded for 30 min with 5 μM Rhod-FF-AM. Subsequently, cells were subjected to de-esterification over 15 min. During this time the BH4 domain peptides were introduced into the cells using the *in situ* electroporation technique^60^. Fluorescence-intensity changes in mitochondria were analyzed with custom-developed FluoFrames software. For each individual trace, the relative change of fluorescence (ΔF/F) was calculated. ΔF/F equals [F_t_-F_0_/F_0_], with F_0_ denoting the fluorescence before stimulation with caffeine and F_t_ the fluorescence at different time points after caffeine stimulation. Subsequently, relative mitochondrial [Ca^2+^] changes were quantified as the area under the curve of the various Ca^2+^ traces.

### Statistical analysis

Two-tailed student's t-tests were performed when two conditions were compared. When comparing three conditions a one-way ANOVA with Bonferroni's multiple comparison test was performed. * indicates significantly different results (p<0.05). Exact p-values are indicated in the figure legends, where available.

## Author Contributions

The study was conceived and originally designed by T.V., H.D.S., J.B.P. and G.B. with additional input from E.V. and L.Ma. for molecular modeling, J.T. and N.N.K. for MAPPIT and hippocampal neurons, respectively. T.V., E.D., I.L., H.I., E.L. and G.M. performed the experiments. T.V., E.D., L.Mi., L.L., H.D.S., I.L., E.L., H.I., G.M., J.T., L.Ma., N.N.K., J.B.P. and G.B. analyzed, interpreted and/or discussed the data. T.V. and G.B. drafted the manuscript. All authors critically revised the manuscript and approved the final article.

## Supplementary Material

Supplementary Information Supplementary Information
