# Supplementary material for: Ryanodine receptors are targeted by anti-apoptotic Bcl-XL involving its BH4 domain and Lys87 from its BH3 domain
Source: Sci Rep. 2015 Apr 15;5:9641. doi: 10.1038/srep09641 (PMC4397538; doi:10.1038/srep09641)
Supplement: Supplementary Information [file srep09641-s1.pdf]

Ryanodine receptors are targeted by anti-apoptotic Bcl-X_L involving its BH4 domain and Lys87 from its BH3 domain

Tim Vervliet[1], Irma Lemmens[2], Elien Vandermarliere[3], Elke Decrock[4], Hristina Ivanova[1], Giovanni Monaco[1], Vincenzo Sorrentino[5], Nael Nadif Kasri[6], Ludwig Missiaen[1], Lennart Martens[3], Humbert De Smedt[1], Luc Leybaert[4], Jan B. Parys[1], Jan Tavernier[2], Geert Bultynck[1*]

Affiliations

1 KU Leuven, Laboratory of Molecular and Cellular Signaling, Department of Cellular and Molecular Medicine, B-3000 Leuven, Belgium

2 University of Gent, Cytokine Receptor Lab, VIB Department of Medical Protein Research, B-9000 Gent, Belgium

3 University of Gent, Computational Omics and Systems Biology Group, VIB Department of Medical Protein Research, B-9000 Gent, Belgium

4 University of Gent, Physiology Group, Department of Basic Medical Sciences, B-9000 Gent, Belgium

5 University of Siena, Molecular Medicine Section, Department of Molecular and Developmental Medicine, and Interuniversitary Institute of Myology, 53100 Siena, Italy

6 Radboud University Medical Center, Donders Institute for Brain, Cognition and Behaviour, Department of Cognitive Neuroscience, Department of Human Genetics, 6500HB Nijmegen, The Netherlands

* Corresponding author:

Name: Geert Bultynck

Address: Laboratory of Molecular and Cellular Signaling,
Department of Cellular and Molecular Medicine, KU Leuven
Campus Gasthuisberg, O&N I Herestraat 49 - bus 802, B-3000 Leuven Belgium

e-mail: geert.bultynck@med.kuleuven.be Telephone: +32 16 330215

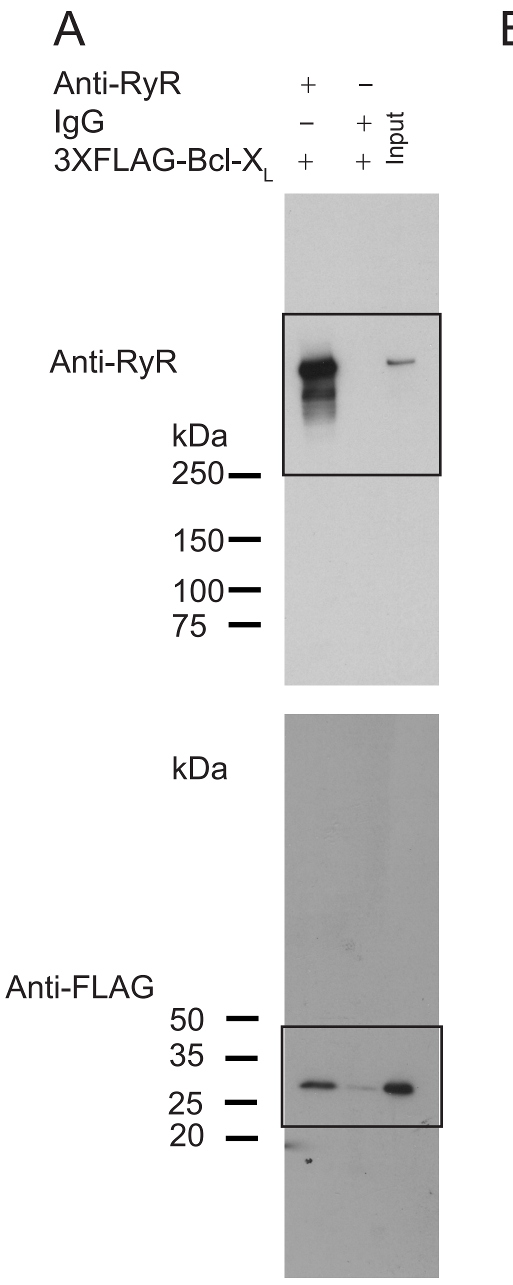

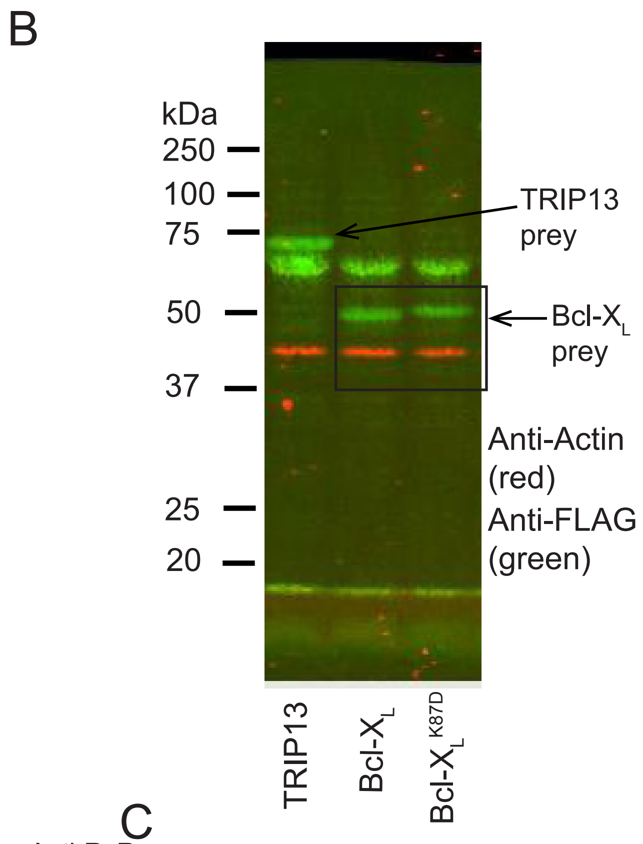

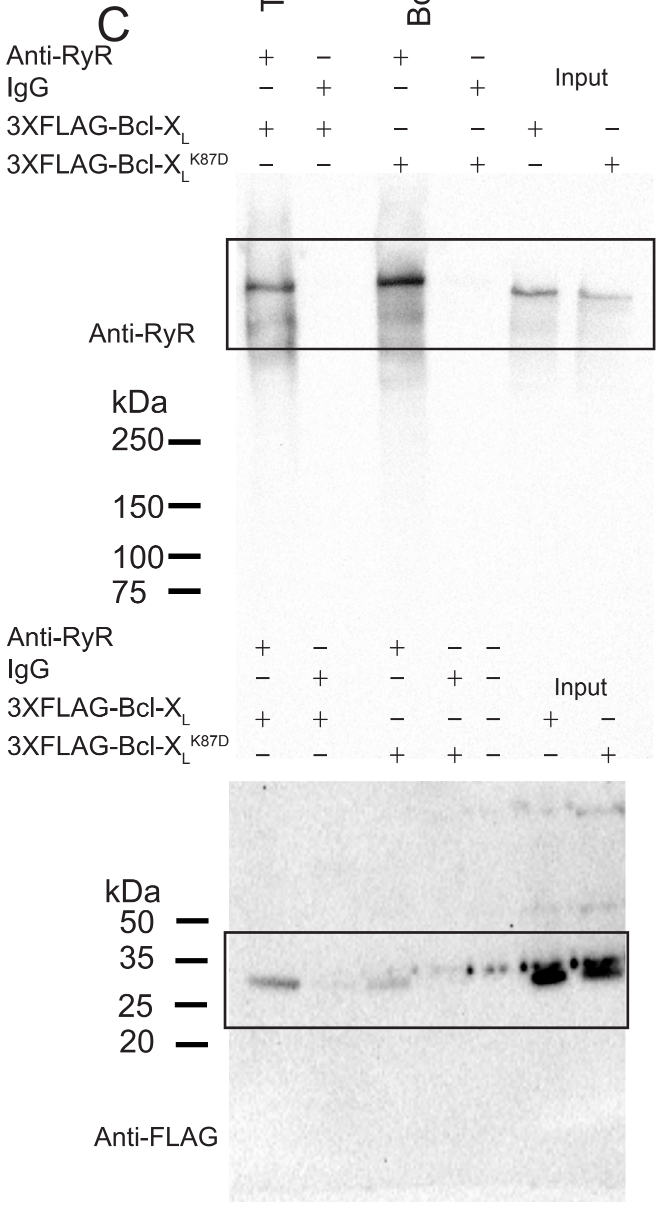

Supplementary Figure 1: Uncropped western blots used in figure 1 (A) and figure 3 (B, C). The boxes indicate where the figure was cropped in the main text. In panel B an additional lane was shown in which a TRIP13 prey was transfected. This protein migrates at a different molecular weight than the Bcl-X_L preys, confirming that the anti-FLAG positive signal at 50 kDa are the the Bcl-X_L preys. This lane was excluded from the main figure as it was not discussed in the main text.

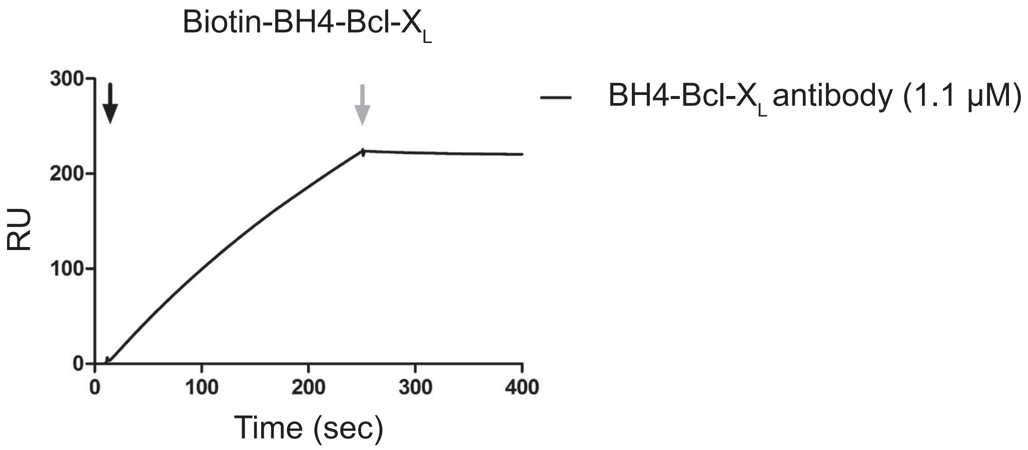

Supplementary Figure 2: SPR sensorgram showing binding of a BH4-Bcl-X$_L$ recognizing antibody to the immobilized biotin-BH4-Bcl-X$_L$. The trace was corrected for background binding by subtracting binding to the scrambled biotin-BH4-Bcl-X$_L$. The black arrow indicates the start of the association phase; the grey arrow indicates the start of the dissociation phase.
